# Gleanings in Science and Medicine

**Published:** 1893-04-29

**Authors:** 


### Gleanings in Science and Medicine.

The most popular sport in Samoa is that of pigeon-chasing, which is entered into with the greatest keenness and zeal, and for the carrying out of which vast preparations are made. Open spaces in the woods are specially designed and kept for this sport, and are called the Tia. The expeditions often last a month, and dwelling-huts are run up in these clear spaces for the accommodation of the chiefs. The winning sportsman is he who entraps the greatest number of wild pigeons, and this is effected in a curiously involved manner. Each chief is the possessor of a tame bird of that kind, the leg of which is attached by to a stick a long line of string. At the end of the stick, in front of this pigeon, and depending from it, is a net-bag, which opens in its upward passage. The chiefs simultaneously let their tame pets fly on high, when the wild prey are curiously attracted towards them, and getting entangled in the nets, are brought downwards in this manner. The birds thus lured are victims of sport alone, and are not destined for purposes of food, as a plentiful supply is carefully laid in for these expeditions, consisting of pigs, native fruits, bread fruit, &c.

Very pathetic, and quite unaccountable, too, are the accounts which reach us of the overflowing fulness of the Morgue, at Paris. Spring is not a season suggestive of despair or hopelessness, but for some unexplainable reason it seems to have inspired with such feelings many miserable inhabitants of the French capital. The first week in April (says a special correspondent) saw no less than fifty-seven corpses brought to this said House of Identification, on the banks of the Seine. Indeed, the resources of the officials which are attached to the Morgue are still taxed to their uttermost to meet the demands thus laid upon them, for the place is full to overflowing. Nowadays few bodies are actually interred without identification. Much has been done to prevent their being ultimately consigned namelessly to the grave. Every chance and opportunity is given to enable friends to recognise the unfortunate victims who are brought to this public mortuary. The bodies are first photographed, and then frozen, so that they can be retained without infringing the laws of sanitation for a given number of days. Curious and tragic are the stories which are current about La Morgue, "La Bague Fatale" being the least universally known. This ring was one of evil omen, for it was found again and again on the hand of some member of the same family, and always in possession of one whose end was tragic. It is said to have appeared at La Morgue five times, to have been claimed each time, to appear again on the finger of some fresh victim.

Science is being brought to bear now on the improved shoeing of horses' hoofs, with as great an advantage to the animals as to their owners. A well-shod horse is capable of twice the work of an ill-shod one, and it is with a view to the improved condition of the existing order of things that a scheme is being formulated in the South of England to start competitions in this art. In the actual harnessing of horses we see a marked improvement as time goes on; indeed, the heavy trappings of past ages are preserved as curiosities—the horse of to-day is not equally trammelled—but by no means can it be said that perfection has been arrived at in our harnessing. We still universally use blinkers on our harness bridles; America has for a great part discarded them as unhealthily hot for the horse's eyes, and consequently injurious to their vision. Much of the short-sightedness which we observe is possibly traceable to these blinkers themselves. We heard lately of a case where the master, after consulting an oculist, ordered a pair of spectales to be made for his horse, which gave perfect satisfaction, the animal giving every evidence in a marked way of his appreciation of the new facility to sight. Some tramways in New York have surmounted the evil of this overheating of horses' eyes through the closeness of the blinkers, by so constructing the blinds that they stand out straight, thus permitting of a free play of air, and also preventing the horse seeing the whip, or anything which is actually behind it.

